# Mitigating Challenges of the Neutral Oxygen Evolution Reaction: The Undervalued Importance of Electrolyte Engineering

**DOI:** 10.1021/jacsau.5c01059

**Published:** 2025-12-08

**Authors:** Erica D. Clinton, Juliana Bruneli Falqueto, Thomas J. Schmidt, Emiliana Fabbri

**Affiliations:** † PSI Center for Energy & Environmental Science, Paul Scherrer Institute, 5232 Villigen PSI, Switzerland; ‡ Institute for Molecular Physical Science, ETHZ, 8093 Zürich, Switzerland

**Keywords:** oxygen evolution reaction, neutral pH, electrolyte, buffer, water splitting

## Abstract

Water splitting in quasi-neutral electrolytes (5 ≤ pH ≤ 10) offers advantages over highly alkaline or acidic electrolytes by improving operational safety, simplifying maintenance, and enabling the use of diverse natural water sources. Such electrolytes suggest promise for large-scale applications, especially considering their widened catalyst scope. However, kinetically restrained oxygen evolution reaction (OER) catalysts exhibit decreased electrochemical performance in these conditions, marked by reduced activity and increased overpotential. This problem has been primarily addressed by exploring catalyst design without proper consideration of the electrolyte; its role is often undervalued and overlooked. Electrolyte parameters, including ionic species, pH, physicochemical properties, etc., profoundly impact the OER. If not properly chosen, catalyst surface interactions, local pH swings, and factors like mass transport can become nonideal. Therefore, quasi-neutral electrolytes must be methodically selected for a given system. In this perspective, we present the challenges faced in quasi-neutral electrolytes and emphasize points of consideration for quasi-neutral OER electrolytes by systematically reviewing the literature. First, we explore buffers and local pH, and the surface interactions between the catalyst and the electrolyte. Next, we discuss electrolyte additives and their promise to enhance the OER by altering the electric double layer and hydrogen bonding environment. Lastly, we address the critical role of mass transport through the lens of physicochemical properties and external parameters. Overall, a strategic approach, encompassing an informed choice of an electrolyte and modification of its properties, is suggested for enhancing OER performance to drive quasi-neutral electrochemical water splitting innovations.

## Graphical abstract

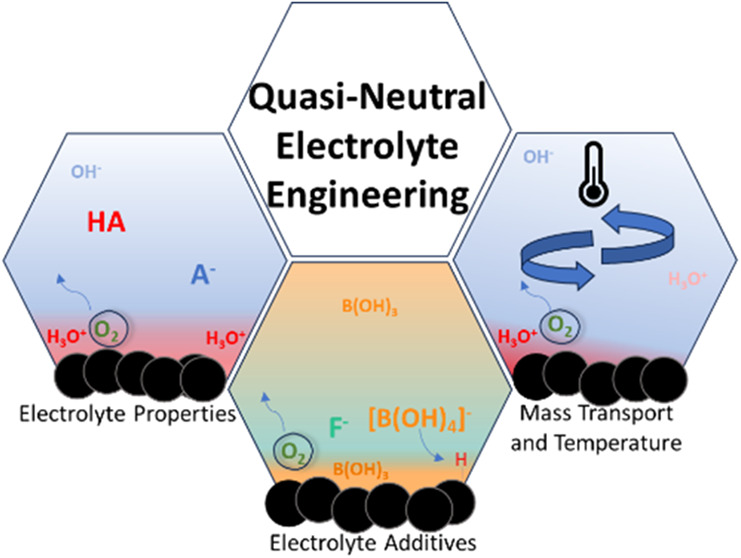

## 1 Introduction

Global energy consumption is projected to climb by an average of 3.4% annually for the next few years.^1^ With an ever-growing energy demand, switching from carbon-based fossil fuels to a green energy source is imperative to slow down and mitigate climate change. One promising contender to alleviate fossil fuel emissions is green hydrogen. Green hydrogen can be produced sustainably through water electrolysis, utilizing water as the reactant.^2^ Leveraging on the hydrogen evolution reaction (HER) and the oxygen evolution reaction (OER), water electrolysis produces oxygen as its only byproduct when using renewable energy sources like solar, wind, or hydropower.^3^ Thus, this solution offers minimal environmental impact.

Within industry, the production of electrolyzer-produced hydrogen is dominated by two technologies: the alkaline water electrolyzer (AWE) and the proton exchange membrane water electrolyzer (PEMWE).^4^ The AWE is currently the most mature technology and is suggested to be the most commercially viable option.^5^ That said, it is not without problems. These technologies use corrosive electrolytes that pose hazards for workers and increase the need for maintenance. Moreover, electrocatalysts are also limited to corrosion-resistant materials such as expensive and scarce noble metals.^6–8^ Currently, a shift toward replacing alkaline (pH > 10) or acidic mediums (pH < 5) with nearly neutral (5 ≤ pH ≤ 10) electrolytes and water from naturally occurring sources has been a topic of much interest.^9–11^ This would enable hydrogen production anywhere a water source is present: seawater, wastewater, and brackish groundwater. It is proposed that by delocalizing hydrogen production into small-to-moderate-scaled plants using nearby water sources, the complexity, cost, and emissions that arise from creating a large-scale transportation network or dispersing generated fuel could be lessened.^12,13^

While a nearly neutral environment is enticing, it can be plagued by a multitude of problems. For one, natural or wastewater sources contain other contaminants such as chloride, magnesium, and calcium in abundance. For example, the presence of chloride in seawater can lead to the chlorine evolution reaction (ClER), which unfavorably competes with the OER.^14^ This side reaction not only reduces faradaic efficiency but can also generate toxic chlorine gas and other corrosive byproducts that accelerate electrode degradation.^15^ Another major problem is that, in general, a catalyst in a nearly neutral environment presents lower electrocatalytic activity and higher overpotentials than its alkaline counterpart.^16^ Such problems become evident in pure water anion exchange membrane water electrolyzers (AEMWE), where catalyst dissolution and membrane instability originate from local pH shifts, resulting in decreased electrochemical performance.^17^

Currently, to address the general problems in quasi-neutral pH electrolytes, emphasis has been put on catalyst and membrane design. Many types of electrocatalysts have been probed for the OER and HER, including precious metals, earth-abundant, and transition metal-based catalysts. These catalysts are further refined for electrocatalytic performance in quasi-neutral environments through various methods, such as topotactic conversions and modification of crystal/electronic structure^11,16,18^ as well as functionalization of electrocatalyst surfaces.^19–21^ While the composition of the catalyst and its design are integral to the electrocatalytic performance, the electrolyte has a significant impact on the electrode/electrolyte interface and, consequently, on electrocatalytic processes. For example, the electrolyte dictates intermediates and their stabilization, the reaction pathway, the electronic field at the interface, the hydrogen bonding network of water, mass transport, etc.^11,22–26^ Understanding its role is paramount for optimizing the nearly neutral OER/HER. In different quasi-neutral electrolytes/buffers, the HER has been extensively discussed.^27–32^ Research also extends to direct seawater electrolysis,^14,33^ but elucidating electrolyte effects in such a complex environment is difficult. The conversation around understanding the fundamental basis of nearly neutral electrolytes and choosing an appropriate neutral OER electrolyte is much quieter, which is the kinetically sluggish and more difficult half-reaction.^24,34^

Electrolyte engineering is a broad term used to describe altering the properties of an electrolyte to achieve a desired effect. In 2017, Shinagawa et al.^25^ changed electrolyte properties to minimize concentration overpotential, which became their definition for electrolyte engineering. Moreover, Komiya et al.^35^ engineered their electrolyte for neutral-based electrolysis by mixing different buffers and adding KCl to enhance conductivity and performance. The term electrolyte engineering will be used in this work to describe inducing changes to the electrolyte to alter the interfacial properties and elevate the OER. In this perspective, we discuss the challenges of the nearly neutral OER and explore the impact of altering various electrolyte parameters through current research performed in simplistic and controlled environments. While doing so, we highlight important key electrolyte parameters for quasi-neutral pHs. We start by considering the mechanistic principles of the OER between the nearly neutral range of 5 ≤ pH ≤ 10, and then briefly examine the impact of local pH on the electrocatalytic system. Following this, we discuss the literature that has reported electrolyte engineering in quasi-neutral environments for the OER. This includes covering the intrinsic nature of buffering systems and their interactions with the catalyst, additives that change the electric double-layer dynamics at the electrode interface, and external parameters to encourage mass transport. Lastly, we suggest factors that must be considered for research and system design.

Crucially, even though this perspective is centered on the OER, the principles of electrolyte engineering presented herein can address a variety of electrochemical reactions in quasi-neutral conditions. It can be applied to many reaction systems, including the OER, HER, and their reduction/oxidation counterparts, formic acid oxidation and CO_2_ reduction. For example, the HER consumes H^+^ or produces OH^–^, resulting in local alkalinity. Buffer species can help counteract this problem in quasi-neutral electrolytes if the environment is carefully engineered. More details can be found in an article from Shinagawa and Takanabe,^30^ where they analyze electrolyte engineering for the HER (around pH 5) using a microkinetic lens on a Pt catalyst. Therefore, while this perspective focuses on the kinetically sluggish OER, the overarching ideas regarding quasi-neutral electrolyte optimization can be applied more broadly to other quasi-neutral electrocatalytic systems.

## 2 How do Quasi-Neutral Environments Affect the Mechanism and Interfacial Properties of the Electrocatalytic OER?

Understanding the mechanism of the OER in nearly neutral environments is imperative for OER optimization: an electrolyte controls many parameters around OER performance including the electric double layer (EDL), hydrogen bonding networks, transition states, water clustering and orientation, ionic interactions, surface coverage, electric fields, oxidation states, and concentration gradients.^36^ In Section 2.1, we explore the current fundamental understanding of the OER in a quasi-neutral electrolyte (5 ≤ pH ≤ 10), highlighting reactant switching between the acidic and basic pathways that can be triggered by pH and diffusional limits. Then, in Section 2.2, we delve into the persisting problems of the OER that arise with the utilization of a nearly neutral electrolyte, such as local pH (local interfacial pH) swings and catalyst dissolution.

### 2.1 What is the Mechanistic Understanding of the Quasi-Neutral OER?

The mechanism of the OER is not fully understood due to the complexity of a multistep four-electron transfer. While numerous mechanisms have been proposed for the OER,^37^ the two most referenced mechanisms are the adsorbate evolution mechanism (AEM) and the lattice oxygen evolution mechanism (LOEM), as presented in Figure 1a.^38–42^ In quasi-neutral environments, the acidic and basic pathways for AEM-like or LOEM-like mechanisms can be simultaneously active, but one pathway tends to be more dominant.^25,43,44^

**Figure 1:**
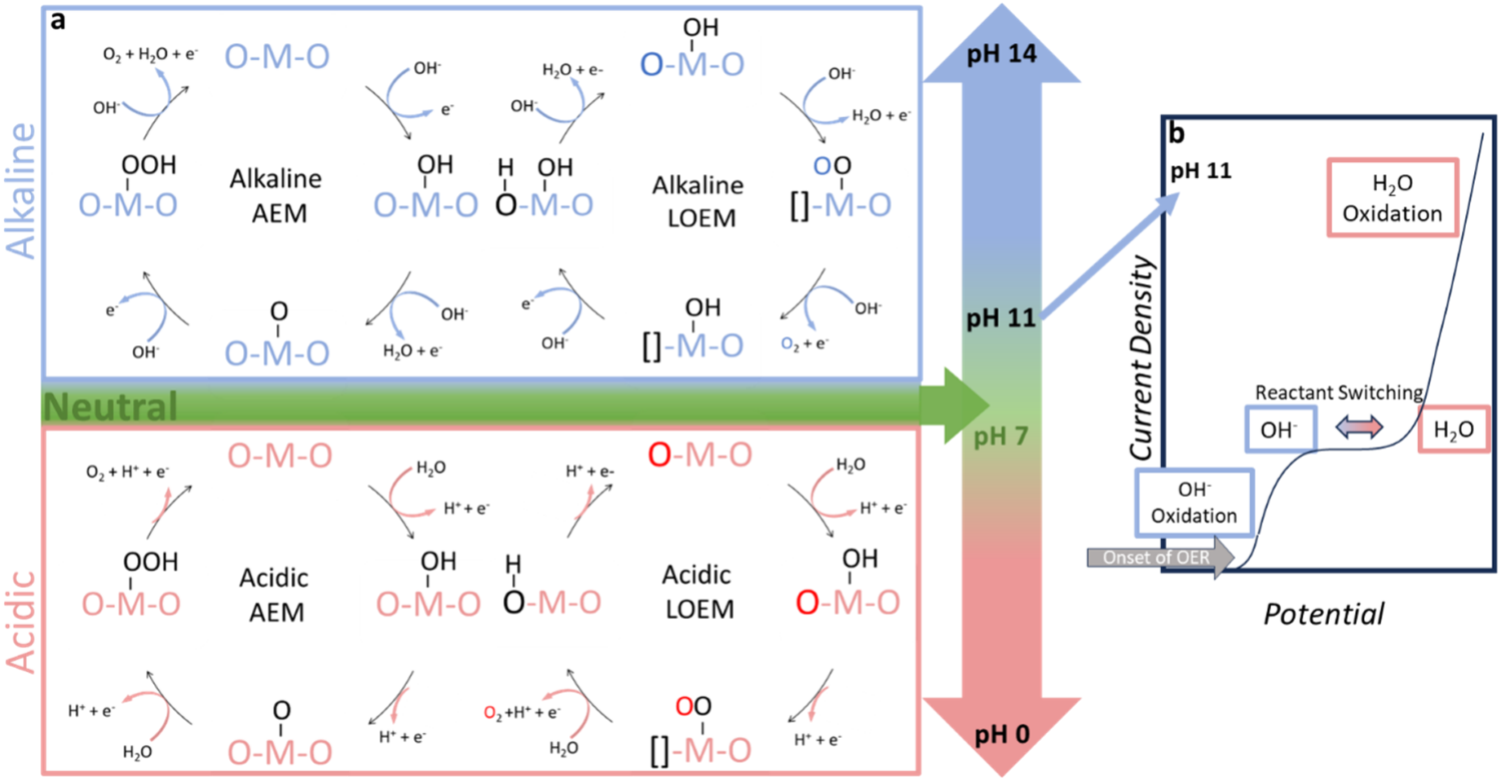
Neutral oxygen evolution reaction mechanistic pathways. (a) The proposed OER pathways depend on the catalyst type and local electrolyte pH; the upper panel shows basic conditions, and the lower panel shows acidic conditions. (b) Reactant switching between OH^–^ and H_2_O oxidation in unbuffered solutions is seen around pH 11, where a plateau in current (as seen in the represented potential versus current density curve) occurs when interfacial OH^–^ is consumed.^44^ Current density eventually increases with applied potential as H_2_O is oxidized.

The switching of acidic and basic pathways occurs in nearly neutral electrolytes as both reactants are present. Whether it follows the acidic or basic pathway is determined by the current density and properties of the electrolyte, including diffusion. At low currents below 20 mA cm^–2^, it has been suggested that mass transport is not an issue, and both mechanistic pathways are present.^44^ Strikingly, within these current confines, the neutral OER can outperform alkaline and acidic environments.^43^ However, at high currents in neutral media, the alkaline route quickly exhausts the interfacial OH^–^ ions, leaving H_2_O as the primary reactant. This means that the acidic pathway is pursued, and the production of H_3_O^+^ greatly increases, disrupting the pH at the surface of the electrode (local pH). This forms a large concentration gradient, for which the consequences will be discussed in Section 2.2. A buffering electrolyte (discussed in Section 3.1) can be used to control the switch to the acidic pathway and mitigate the concentration gradient.^44^ If buffering is not desired, complete reactant switching in salt-based electrolytes, regardless of electrode identity, can be observed around pH 10–11; two-step anodic events, one related to OH^–^ oxidation and the other to H_2_O oxidation, can be visualized in linear sweep voltammetry (LSV) as seen in Figure 1b.^25,44,45^

The AEM consists of four proton-coupled electron transfer steps (PCET) (Figure 1a left). These steps are traditionally concerted in nature (also called concerted proton and electron transfers, CPET). Importantly, for the AEM, the metal redox center describes the electronic states near the Fermi level.^46^ As a result, the metal valence band interacts with the intermediates, and their energies scale together. This scaling relationship creates a minimal overpotential necessity of 370 mV.^47^ It should also be stated that these steps in nearly neutral (and also other pHs) are not necessarily concerted and can be more complex than the traditional AEM. One highly cited example is a self-healing catalyst based on Co, studied in phosphate and salt-based electrolytes at pH 7; Surendranath et al.^48^ investigated Co-Pi (Cobalt phosphate) and proposed a PCET mechanism involving Co^2+/3+^ and Co ^3+/4+^ species. They suggested a mechanism involving a rapid, reversible one-electron transfer and a phosphate-mediated proton transfer in a pre-equilibrium step followed by a chemical rate-limiting step. In contrast, LOEM-proceeding catalysts (Figure 1a right) have Fermi levels characterized by oxygen, and at least one proton and electron transfer step is decoupled.^49^ This mechanism dismantles the AEM scaling relationship by generating oxygen through direct O–O coupling rather than the formation of OOH*. LOEM has been largely observed in alkaline and acidic media.^38,40,50,51^ Interestingly, the term LOEM is quite sparse in quasi-neutral OER literature. While it is pointed out in different papers and reviews,^11,38^ Lai et al.^9^ suggested that it has not been experimentally confirmed in neutral conditions. This information is indeed scarce, but not absent; one study from 2020 by Zhang et al.^52^ noted that RuIrCaO_*x*_ in 0.5 M KHCO_3_ (pH ∼ 7) participated in the LOEM, as observed through *in situ* XAS and isotope studies. Metal sites appear to be the more common active sites in neutral environments, and catalysts undergo PCET. Despite the lack of substantial evidence, the LOEM should not be excluded, as the intrinsic nature of certain catalysts provides the opportunity for such a mechanism. As shared by Binninger et al.,^53^ from a thermodynamic viewpoint, any metal oxide, regardless of environmental pH value, innately holds thermodynamic instability of the oxygen anion in the lattice, indicating that the LOEM can be favored at OER potentials. Such an example is a La_1–*x*_Sr_*x*_CoO_3_ (LSC) perovskite, which undergoes the LOEM in alkaline media and is theorized to participate in the LOEM in neutral buffer, although this is somewhat debated.^19,20,38,39^ In this case, the major hiccup for the LOEM in neutral is that the rate-determining step is suggested to involve proton transfer; the lack of OH^–^ species available to act as a base can be detrimental to the OER kinetics. Overall, Kim et al.^39^ suggest that as AEM and LOEM are thermodynamically and kinetically coupled, both mechanisms occur in conjunction, especially for perovskite catalysts. The predominance of an AEM-like or LOEM-like mechanism is highly dependent on the characteristics of the catalyst and its covalency with oxygenated intermediates.

In general, according to Koper,^47^ the preference for CPET or decoupled PCET is influenced by the lowest activation energy pathway (including reorganization energies) and the catalyst-intermediate interaction strength. Catalysts with stronger intermediate interactions and lower activation energy for concerted versus decoupled steps likely undergo CPET. In principle, these catalysts do not show pH dependence on the reversible hydrogen electrode (RHE) and have Nernstian behavior. That said, it is critical to emphasize when considering multistep mechanisms that a nonzero mV/pH slope does not necessarily rule out a CPET; the quasi-equilibrium steps before the RDS can influence the slope. Conversely, intermediates weakly engaging with the catalysts show increased dependence on the electrolyte for stabilization, and the mismatch in electron transfer kinetics and H^+^ affinity can lead to pH dependence observable on the RHE scale.^38,47,51^ Importantly, the p*K*_*a*_ of a key intermediate can significantly alter the required overpotential, highlighting that catalysts with decoupled mechanisms have an optimal electrolyte pH for an improved turnover. Thus, for any catalysts undergoing decoupled transfers, the pH of the electrolyte is vital for the OER performance.

### 2.2 What are the Persisting Problems in Quasi-Neutral Electrolyte for the OER?

Due to the scarcity of OH^–^ and the abundance of H_2_O molecules near pH 7, the acidic path is proposed to dominate the reaction in unbuffered conditions.^44^ Consequently, the interfacial pH can become susceptible to drastic pH swings, paving the path for problems like accelerated degradation, massive concentration gradients, and altered adsorbate binding energies (Figure 2c). Obata et al.^54^ used *in situ* fluorescence in stagnant 0.5 M K_2_SO_4_ (Figure 2a) and 0.1 M K-phosphate (Figure 2b). After 5 min at 1 mA cm^–2^, a pH change greater than 1.75 (maximum detection range available via *in situ* fluorescence pH sensor foil) was found to span approximately 1 cm from the electrode surface. This substantial pH shift necessitated increased overpotential to match the current densities seen in phosphate buffer. Furthermore, Yokoyama et al.^55^ used rotating ring disk electrode (RRDE, IrO_*x*_ ring, Pt disk) LSV measurements (0.6–1.7 V vs. SHE). They demonstrated a staggering drop of 3.6 pH units in a neutral Ar-saturated NaClO_4_ solution. It is crucial to recognize that these extreme local pH fluctuations can occur at any nearly neutral pH; substantial local pH fluctuations occur on the higher end of the quasi-neutral regime at very low currents despite the presence of more OH^–^. Fornaciari et al.^43^ demonstrated that even with an initial pH of 9, simulated pH swings with current densities <3 mA cm^–2^ can result in a local pH drop below pH 4. While these works allow a glimpse into the pH change at a measurable distance from the surface, it can be assumed that the actual electrode interface undergoes even more extreme pH deviations.

**Figure 2:**
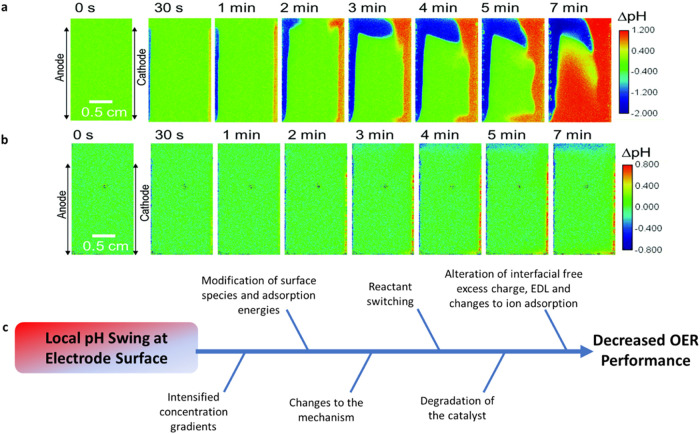
Local pH fluctuations. (a–b) *In situ* fluorescence monitoring of local pH during electrochemical water splitting in (a) unbuffered 0.5 M K_2_SO_4_ and (b) buffered 0.1 M K-phosphate starting from pH 7. Panels (a) and (b) are reproduced from ref^54^ via Creative Commons CC BY 3.0 license. (c) The changes to the OER induced by local pH swings can lead to decreased electrochemical performance.

Harsh local pH fluctuations at the electrolyte/electrode interface, stemming from an electrolyte’s inability to manage interfacial acidity due to insufficient buffering capacity, can induce catalyst instability. Takashima et al.^56^ found that below pH 9 (and especially in more acidic environments), MnO_2_ catalysts show instability that triggers disproportionation of Mn^3+^ into Mn^2+^ and Mn^4+^ states. This dramatically devastates the OER performance and raises the overpotential. This highlights that extreme pH swings could render pH-sensitive catalysts like MnO_2_ ineffective if a proper buffering electrolyte is not chosen.

Furthermore, beyond local pH-induced instability, thermodynamic-based unsteadiness in neutral conditions has been noted for perovskites.^39^ Han et al.^57^ discovered that in a pH 7 solution (mixed NaH_2_PO_4_/Na_2_SO_4_/NaOH), perovskites amorphized and leached A-site doping cations more quickly when exposed to neutral environments if their O p bands were closer to the Fermi level. On the other hand, those with O p bands away from the Fermi level not only leach A-sites slowly but also B-site cations at appreciable current densities. This is important as B-sites are considered the active sites for the AEM and thus OER activity drops (along with possible effects from electrolyte deactivation). To minimize dissolution where self-reconstruction seems to be lacking, Pourbaix diagrams can be utilized, which illustrate the thermodynamic stability as a function of pH and applied potential. This is especially relevant as achieving appreciable current densities in quasi-neutral electrolytes often necessitates higher overpotential, putting severe oxidative stress on the catalyst and accelerating degradation. Despite Pourbaix diagrams being a useful tool, they do not consider the full nature of a specific electrolyte and cannot be solely used to determine catalyst stability in a system.

Typically, in more extreme alkaline conditions, leaching from perovskites is not as troublesome as it is in neutral pHs; the presence of the LOEM can encourage the formation of an OER active phase, drastically improving a catalyst’s performance. This process occurs when metal cations are dissolved and oxidized or recombined with electrolyte OH^–^ to form a newly reconstructed oxy­(hydroxide) surface.^39^ One example is from Fabbri et al.,^40^ where *operando* X-ray absorption spectroscopy (XAS) showed that perovskites like Ba_0.5_Sr_0.5_Co_0.8_Fe_0.2_O_3‑δ_ (BSCF82) generate a dynamic self-forming metal oxy­(hydroxide) active phase in hydroxide-rich environments, improving OER performance. That said, self-reconstruction is quite sensitive to the environmental pH (most notably here), defects (which alter the electronic structure to encourage reconstruction), and applied potential.^58–60^ In neutral environments, negligible activity-enhancing self-reconstruction has been noted. This hinders the improvements seen in alkaline environments, and worsening performance over time can occur.^38^ Kim et al.^39^ examined Ba_0.5_Sr_0.5_CoO_3‑δ_ (BSC) and BSCF through Fourier transform extended X-ray absorption fine structure (FT-EXAFS) and noted minimal metal oxy­(hydroxide) layers formed. Thus, self-reconstruction-induced performance benefits become minimized in quasi-neutral pHs, suggesting that activation in higher pHs is necessary for some catalysts. That said, it is currently unclear if active phase formation is limited by mechanistic principles or the decrease of OH^–^ in quasi-neutral electrolytes.

In addition to catalyst dissolution and instability, mass transport at the electrode surface can be crippling to a neutral-based OER system. Not only does the diffusion of OH^–^ impact the OER, but in buffering solution, the buffering species also must be transported and are thus important for mass transport phenomena.^25^ As buffering species are not reactants, their main job is to transfer protons away from the surface and for their unprotonated counterparts to go to the surface, without causing issues with specific adsorption. The efficiency of such transfers depends on electrolyte parameters. For example, Shinagawa and colleagues^61^ demonstrated for the HER using a Pt-based catalyst that increasing the concentration of phosphate buffer resulted in the need for higher overpotentials due to the diffusion of protonated buffer species. While this is much less investigated for the OER in neutral conditions (discussed later), properties like molarity, mean activity coefficient, size, and viscosity of the chosen electrolyte directly manipulate mass transport and must be considered for optimization: electrolyte engineering.

## 3 Buffering Capacity and Electrolyte/Electrode Systems: What Intrinsic Properties of an Electrolyte Should be Considered?

Salt-based electrolytes such as K_2_SO_4_, NaClO_4_, and KCl can be employed to achieve nearly neutral conditions with high ionic conductivity. However, pH in these solutions can drastically swing during the OER, leading to a cascade of issues, including concentration gradients and thus catalyst instability, as mentioned in the previous section. Additionally, certain salts can induce competing side reactions, such as the ClER, reducing faradaic efficiency and accelerating anode corrosion.^21^ Therefore, choosing a nearly neutral electrolyte requires careful consideration, as its inherent properties influence the interfacial dynamics between the electrolyte and the catalyst. One key issue, pH instability, can be addressed by utilizing buffers as the system’s electrolyte and is examined below.

### 3.1 What are Buffers and How Do They Affect the OER?

Buffer solutions exhibit resistance to pH change; they consist of a weak conjugate acid–base pair that mediates pH fluctuations. This resistance is called buffering capacity. Generally, it is defined by an acid–base conjugate combination that can facilitate pH steadiness within ∼±1 pH unit of the p*K*_*a*_ at a temperature *T*. Buffering capacity has also been used to describe a more mathematically rigorous definition: the derivative of the amount of base or acid added with respect to the change in pH (also known as buffering intensity).^62^ The Henderson–Hasselbalch approximation provides an estimate of buffering action and is a simplification of the law of mass action; it can be used to showcase a buffer’s adeptness to resist pH changes with the addition of a strong base or acid, dilution, or neutral salts.^63^ As organic buffers tend to undergo oxidation with applied potentials, only a small handful of buffers are used for the electrocatalytic OER.^64,65^ The three most common are phosphate, borate, and carbonate buffers, and their room temperature p*K*_a_(s) are shown in Figure 3.^63^ It is well-known that buffers resist pH change best when the system’s pH equals their p*K*_a_. Thus, it is imperative for the OER, due to the production of H^+^ in the acidic pathway, that the pH equals the p*K*_a_ to maximize buffering capacity.^36^ Experimental results for a rotating disk electrode setup indicates that shifting the bulk pH one pH unit from the p*K*_a_ (reducing buffering capacity) can decrease the maximum current density by an order of magnitude.^36^ Additionally, staying around the p*K*_a_ is seen to reduce the OER overpotential,^66^ which was demonstrated by an IrO_*x*_ catalyst in phosphate buffer solution across 1–20 mA cm^–2^.^24^

**Figure 3:**
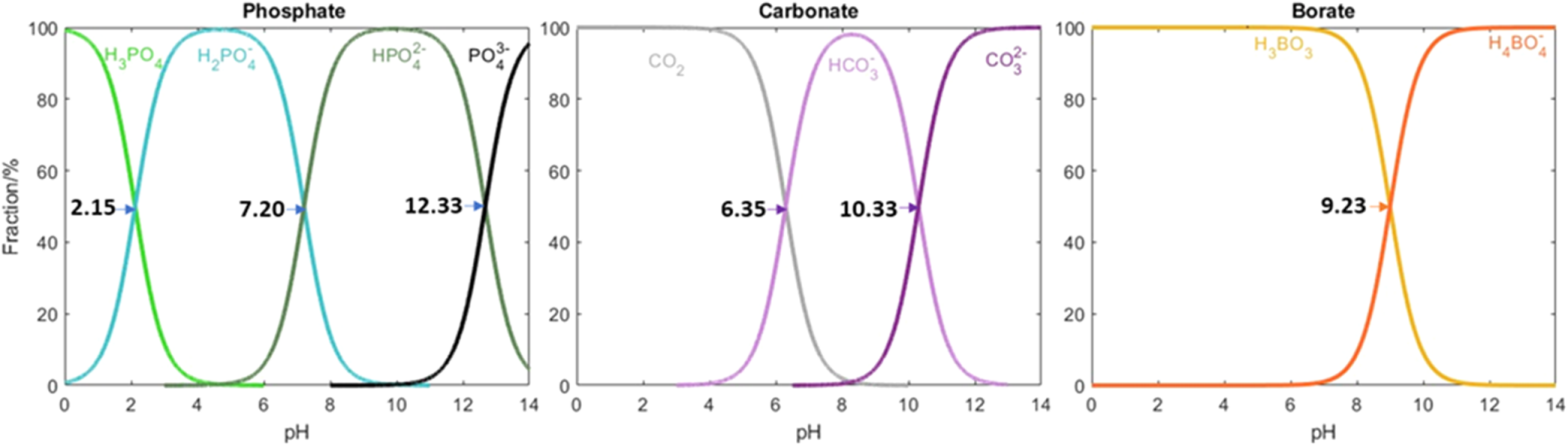
Speciation diagrams of various buffers. Percent fractions at different pHs are shown for phosphate, carbonate, and borate buffers. p*K*_a_s, whose values were obtained from Stoll and Blanchard,^63^ are shown in bold and marked by an arrow. Depending on concentration, borate buffers can have additional species, and this is described in detail by Liu and colleagues.^67^

Mechanistic changes between the acidic and alkaline OER pathways are dependent on electrolyte pH and current density; unbuffered systems at high current densities typically undergo water oxidation via the acidic pathway as OH^–^ is quickly consumed and the local pH drops.^24^ In buffered solutions, Nishimoto and co-workers suggest that because of the inherent nature of buffers to assist in proton removal for pH preservation, which in turn helps maintain the in situ supply of OH^–^, catalysts can use OH^–^ as the primary reactant (basic pathway) if certain criteria are met. This is provided that there is sufficient buffering capacity and mass transport to replenish consumed OH^–^ at the electrode surface and that appreciable current densities do not counteract the buffer’s ability to stabilize local pH.^24^ The hypothesis that OH^–^ is the primary reactant in buffering environments was further backed by a link between the buffering enthalpy and the OER apparent activation energy. This relationship therefore emphasizes that the buffer’s efficiency at replenishing in situ OH^–^ is a kinetically relevant parameter in buffered electrolytes, especially if OH^–^ is the main reactant. The rate constant of OH^–^ (*k*_OH–_) formation results from the buffering equilibrium (eq 1):

1 $$H_{2}O+A^{-}⇌OH^{-}+\mathrm{HA}$$

To explore the interconnectivity of p*K*_a_, *k*_OH–,_ the Tafel slope, and the exchange current density, the group used IrO_2_ in different buffers.^24^ Fitting of experimental data plotted with literature-derived rate constants revealed that the p*K*_*a*_ is proportional to the log of the OH^–^ rate constant (*k*_OH–_) that describes buffering equilibria (Figure 4a). This relationship is important as it suggests that while the availability of OH^–^ is primarily pH-dependent, the p*K*_a_ of a buffering system (at max buffering capacity) determines how the in situ OH^–^ is maintained and how quickly it is replenished. For example, systems with lower p*K*_a_s have a slower local supply rate of OH^–^ from the buffer than those with higher p*K*_a_s (when the p*K*_a_ is equal to the pH). Further insights into this connection were explored by deconvoluting the Tafel slope and exchange current density for various buffering systems, each with its unique p*K*_a_s. At the larger current densities of 10, 20, and 100 mA cm^–2^, the group showed that a Na-phosphate/IrO_2_ interface had lower OER overpotential and a reduced Tafel slope of ∼20 and ∼40 mV dec^–1^, less than Na-carbonate/IrO_2_ or Na-borate/IrO_2_ systems, respectively (again following the condition that the pH was set to the p*K*_a_). This data indicated a critical trade-off relationship; specifically, an improved Tafel slope came at the cost of a lowered exchange current density and a slower *k*_OH–_ as buffer p*K*_a_ (set to pH) decreased. The group proposed that this pattern is most likely a result of pH, as formation of an *O intermediate for the IrO_2_ catalyst is more facile in alkaline environments. Most intriguingly, at low current densities around 1 mA cm^–2^, Na-carbonate/IrO_2_ and Na-borate/IrO_2_ interfaces needed lower overpotential than Na-phosphate/IrO_2_ when the pH was equal to the p*K*_a_ (Figure 4b). This juxtaposes the results at higher current densities. Therefore, when choosing a buffer electrolyte, several factors should be considered: (1) that the p*K*_a_ should be close to the pH that maximizes buffering capacity and minimizes local pH effects, (2) that the buffer be selected for a desired operating current density to maximize OER performance, (3) that a higher p*K*_a_ and higher in situ supply of k_*OH–*_ cannot be the only selection factors; pH can greatly alter the surface species and adversely affect the Tafel kinetics. Comparing the trade-off relationship of the Tafel slope and exchange current density at different pH values (where the buffer’s pH equals the p*K*_a_) can provide valuable insights into minimizing the needed overpotential at a given current density. The applicability is contingent on a stable catalyst and that the mass transport flux is sufficient.

**Figure 4:**
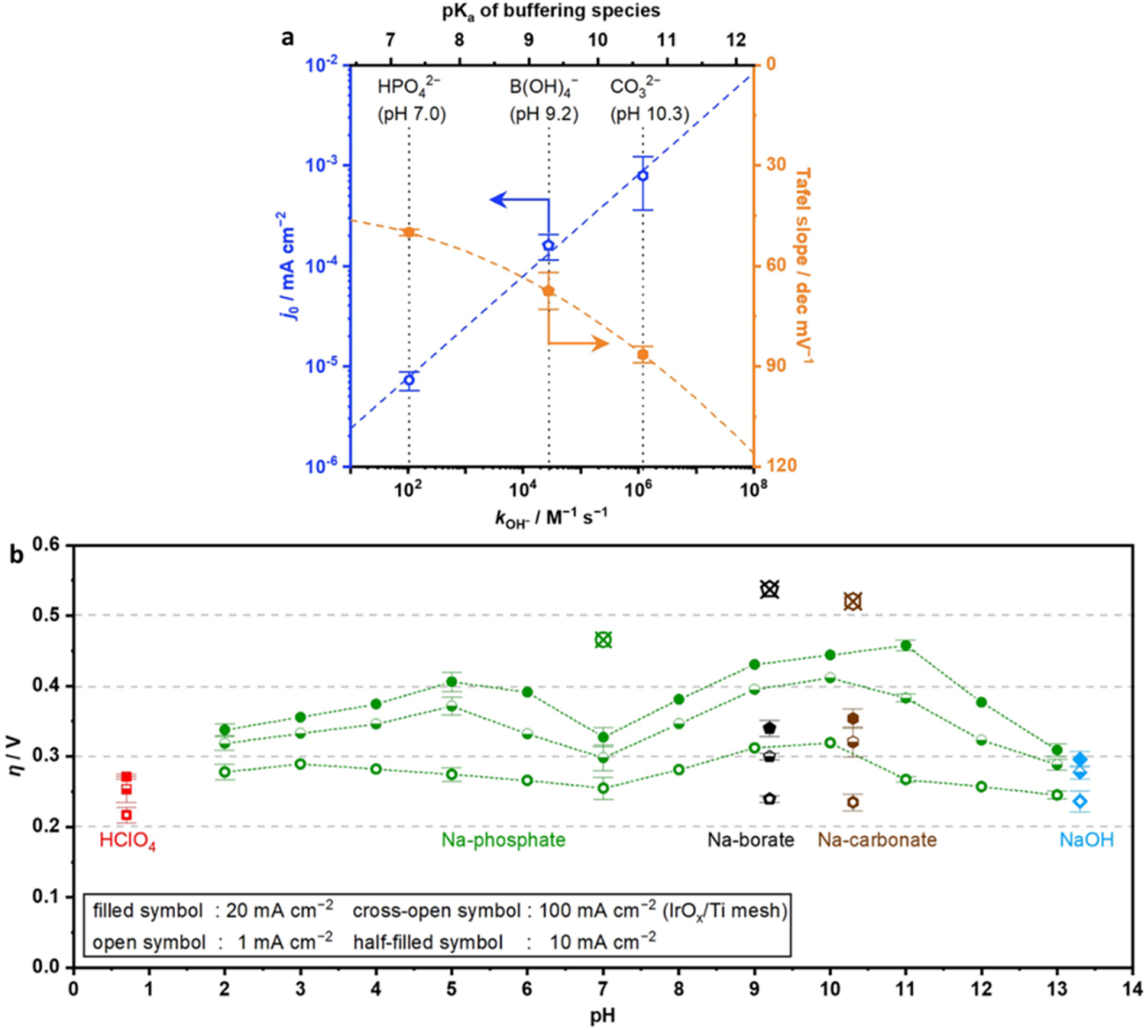
Intrinsic buffer properties and OER performance. (a) The relationship of the exchange current density (*j*_0_), Tafel slope, buffer p*K*_a,_ and the rate constant *k*_OH*–*_ showcases a trade-off relationship between the exchange current density/*k*_OH*–*_ and Tafel slope. Tafel slopes and *j*_0_ were obtained at room temperature via RDE measurements using 1.0 mol kg^–1^ electrolyte solutions and an IrO_*x*_ catalyst on Ti mesh with a scan rate of −1 mV s^–1^. (b) The overpotential needed to achieve current densities of 1, 10, 20, and 100 mA cm^–2^ for an IrO_*x*_ over Ti mesh catalyst measured using a RDE with different electrolytes across various bulk electrolyte pHs. Both panels (a) and (b) are from ref 24 and reproduced with permission copyright @ 2021 Wiley-VCH GmbH.

### 3.2 How Do Buffers Interact with the Catalyst Surface?

Beyond the influence of the p*K*_a_ and stabilizing local pH, the buffer can impact the OER by directly affecting the active electrode surface. One significant interaction that can occur is the deactivation of a catalyst due to buffer species adsorption. For example, phosphate is known to adsorb onto Pt, IrO_*x*_, and RuO_*x*_, an effect extensively seen in acidic environments.^68–71^ To investigate catalyst deactivation resulting from phosphoric acid species relevant to PEM electrolyzers and their membranes, Owe et al.^69^ explored the effects of a 0.5 M H_3_PO_4_ solution at pH 1.2 (across 0–150 °C) on IrO_*x*_ catalysts. These catalysts were anodically made films or nanocrystalline powders. Using an electrochemical quartz crystal microbalance, the study revealed that the total mass change was two times higher in phosphoric acid solution compared to perchloric acid solution at 50 mV , indicating a large degree of intercalation. Additionally, the authors observed a lower current at a given overpotential in phosphoric acid solution than in perchloric or sulfuric acid solution and increasing the concentration of phosphoric acid resulted in a decreased OER rate. Collectively, the authors concluded that the active sites on IrO_*x*_ were blocked by adsorbed phosphoric acid. Moving to the context of nearly neutral pH, Gisbert et al.^70^ examined (both polycrystalline and 111) phosphate adsorption on Pt across different pHs (despite it being a poor neutral and alkaline OER catalyst). Through voltammetric studies and underpotential deposition analysis, they observed that Pt(111) in 5.8 < pH < 11 conditions adsorbs HPO_4_^2–^ around 0.46–0.6 V vs RHE. At higher potentials, HPO_4_^2–^ undergoes another deprotonation, resulting in a change in coordination (bidentate to triple coordination) and the development of irreversible adsorption features. This clearly indicates that buffer species are not always innocent. However, adsorption is not necessarily all bad. While buffer species can inactivate or irreversibly adsorb on an electrode surface, they can also induce positive effects, especially seen in quasi-neutral environments. Komiya et al.^72^ exploited the stabilization effects of phosphate on NiFeO_*x*_ for seawater splitting. The purpose was to minimize catalyst degradation in quasi-neutral environments, especially in the presence of chloride. Phosphate was added to borate buffer (pH 9.2), and the electrode was electrochemically cycled, showing increased stability versus the borate solution alone. This was followed by tests contaminated with potassium chloride; long-term stability tests over 24 h were conducted, coupled with CV measurements before and after, revealing similar stability with or without chloride with the phosphate addition. Komiya et al. also proposed that phosphate pacifies Ni^2+^ from further oxidation (measured through X-ray photoelectron spectroscopy (XPS) and XAS measurements), enhancing the neutral OER through catalyst stabilization. Wang et al.^73^ found similar passivation effects with a homogeneous nickel­(III) complex in phosphate buffer (pH 7.0), where proton transfer was aided but anation suppressed water oxidation. Furthermore, some catalysts that undergo initial conditioning in buffer rely on buffered conditions for enhanced OER postpreparation. Bediako et al.^74^ observed the interaction of borate on Ni-borate thin films at pH 9.2. It was found that in borate electrolyte, borate anions highly covered the catalyst’s active sites; governed by a Langmuir isotherm, an inverse first-order dependence was noted with borate concentration. As a result, borate-free active sites exist in minor equilibrium with borate-bound sites. This means that when borate concentrations were above 30 mM, activation-controlled current density decreased. On the other hand, when borate concentrations were below 30 mM, there was a zeroth-order dependency on borate. Despite borate species from the electrolyte covering the surface and decreasing open active sites, the presence of borate was deemed necessary as it astonishingly demonstrated better OER activity than salt-based electrolytes. The Tafel slope increased from 30 to 100 mV dec^–1^ in NaClO_4_ electrolyte (without borate, pH 8.5). This was suggested not to be a result of pH as NaOH was added during OER to maintain pH. Instead, the origin of the increased Tafel slopes was proposed to be from the transition to a turnover-limiting chemical step directly from the resting state (without preceding electrochemical steps), most likely involving a proton transfer or high turnover-limiting single electron transfer with a high symmetry factor. Consequently, buffer and catalyst interactions are key determinants for OER performance. A balance of inhibitory and facilitatory effects must be thoughtfully examined; buffers help regulate pH, but they also adsorb and dissociate with the catalyst surface, which can significantly alter the OER performance.

### 3.3 Should the Counter Cation of the Buffer Species Be Considered in Electrolyte Selection?

Moving away from the anionic species, the counter cations can also alter the OER through interactions in the EDL. The ion effect, or ion-induced change of reactant/intermediate energy and organization, directly influences the pathway of an electrocatalytic reaction.^75^ While alkaline cation effects have been decently characterized, cation effects in neutral environments are much less defined. Therefore, this discussion will start from the lens of alkaline conditions. Anions are expected to prevail at the anode surface due to electrostatic attraction. That said, studies have also indicated that cations can significantly affect the OER, especially in alkaline environments. In purified electrolytes (pH 13) using a NiOOH catalyst, Michael et al.^76^ noticed that Cs^+^ leads to the highest OER activity in alkaline conditions and K^+^ = Na^+^ = Li^+^ resulted in the lowest activity. While their study did not produce a compelling explanation for the effect, Garcia et al.^77^ suggested that the cation stabilizes active oxygen species, as observed through *in situ* Surface-Enhanced Raman spectroscopy (SERS) using the same catalyst. In contrast, other groups propose that the improvement stems from the robustness of the water solvation shell of small cations and the increased rigidity of the hydrogen bonding network, affecting the transport of reactants.^78^ From a different lens, Görlin et al.^79^ reported that the OER enhancement resulting from larger cations might arise from the difference in the p*K*_b_s of the alkali hydroxides and, thus, is a consequence of pH. Within the quasi-neutral realm, a photoelectrochemical and electrocatalysis study with various catalysts in borate buffer (pH 9, 0.5 M) showed the cation-based impact on the longevity and magnitude of water oxidation currents at 1.9 V vs RHE.^80^ For electrocatalytic water oxidation, Ni-borate, Co-borate, FeOH, and Co_3_O_4_ catalysts showed an increased OER current density when placed in a Li^+^-based electrolyte compared to Na^+^ and K^+^. This is a cation order often considered anomalous for OER in aqueous solutions, especially when compared to alkaline conditions. It is proposed that the proximity of a smaller cation induces the destabilization of the O–H bond (in comparison to Na^+^) along with suppressing the oxygen reduction reaction (ORR).

To extrapolate effects of ionic species into a quasi-neutral environment, the electrode’s isoelectric point (IEP) and potential of total zero charge (PZTC) can serve as a starting point. However, their relevance varies widely among catalysts and conditions.^61,81^ The IEP is defined as the pH where the electrode’s surface charge is zero.^82,83^ This occurs when surface hydroxyl groups, which form in aqueous solutions, are undissociated. If the pH is greater than the IEP, the surface becomes negative and attracts cations to the surface of the catalyst. Conversely, if the pH is less than the IEP, the surface is positive and attracts anions to the catalyst surface. This provides an initial static estimate for ionic interaction with the catalyst surface.

While the IEP describes how the surface charge responds to changes in pH, the PZTC provides a reference point when considering a dynamic electrochemical system and its potential-induced effects. The PZTC is defined as the state where both the electrode’s electronic charge density and the charge from specifically adsorbed species equate to zero. The PZTC is critical; it can alter the performance of a reaction as suggested by Bender et al.^84^ for the alkaline ORR. They found that if ORR conditions for a catalyst were negative relative to the PZTC, then the negative surface invites cations that impact the double layer. This directly changes the stability of intermediate species, modifying the electrochemical performance. Within their study, Pt, Ir, Au, and Ru showed increased ORR performance with cation size. It should also be noted that Bender et al. found the PZTC shifts with high adsorbate coverage, especially for metals like Pt, Ir, and Ru during the ORR. While this example relates directly to alkaline conditions, this concept can be extrapolated to quasi-neutral electrolytes, where little information exists regarding cation effects. Considering parameters such as cation size and the PTZC/IEP is imperative, as it fundamentally alters the electric double layer and thus the catalyst’s electrochemical performance. Therefore, a deeper characterization of cationic effects in quasi-neutral electrolytes should be considered to further engineer the electrolyte for improved OER performance.

## 4 How Can Electrolyte Additives Help to Improve Quasi-Neutral OER Performance?

The interfacial OER environment between electrolytes and catalysts can be further improved by additive introduction: anions, cations, or supporting electrolyte salts (for conductivity purposes). Anion addition can alter the local electric double layer, modify the water hydrogen bonding network, and aid buffer species in proton-relaying services.^26,85,86^ Cations, whose presence in electrolytes can allow for self-healing catalysts in specific systems,^74,87^ can alter the OER active sites and charge transfer. Therefore, diving into ionic additives is imperative for the OER in nearly neutral systems and will be discussed in the next section.

### 4.1 How Do Anions Alter the EDL?

Nearly neutral water oxidation faces challenges beyond local pH fluctuation and the instability/inactivation of electrodes in different electrolytes. The activation of water is strongly dependent on the hydrogen-bonding network of water and plays a key role in the performance of the OER.^75,85^ A disrupted network can increase the population of nonbonded or nonsolvated water molecules, impeding the efficient solvation of H_3_O^+^/OH^–^ species.^86,88^ On the other hand, the extreme rigidity of a water structure can also prevent reorientation or access to the inner Helmholtz plane.^31,88^ Both cases can diminish OER performance. This problem can be addressed by the addition of anions into bulk electrolytes. However, all anions are not created equal. Sui et al.^88^ have pointed out that low-ionic-potential anions, particularly those that are less hydrated and bulkier, can impede the OER by leading to a less structured hydrogen-bonding network. Contrarily, anions with higher ionic potentials can ease the frustration of solvation for H_3_O^+^ and OH^–^ and elevate the OER, reducing the overpotential needed to oxidize water. To derive these conclusions, Sui et al. paired Li^+^ with different anions at pH 7. They then investigated the anion’s impact on (1) physical properties of the electrolyte and (2) the OER performance using Pt foils. To characterize the average H-bonding environment, ^1^H NMR was collected with the following anions: SO_4_^2–^, NO_3_^–^, ClO_4_^–^ or TFSI^–^ [bis­(trifluoromethansulfonyl)­imde]. Results from Sui et al. reveal that bulkier, low-ionic-potential anions like TFSI^–^, have a lower chemical shift, indicating a weakened H-bonding network. This observation was correlated with LSV measurements to understand the effect on the OER; smaller, high-ionic species showed reduced overpotential than their bulkier anion counterparts. Based on these findings, Sui et al. concluded that the anion-induced alterations of the hydrogen bonding network directly impact the thermodynamic stability of water in neutral environments, and that the chemical environment of these species can lead to varying degrees of solvation frustration, leading to the different observed overpotentials. To further gain deeper insights into how anions changed the hydrogen-bonding network, Sui et al. employed wavelength-tunable ground-state femtosecond stimulated Raman spectroscopy (FSRS) to probe the diverse configurations of water molecules (double donor–donor–acceptor (DDAA), donor–acceptor (DA), or nonbonded/nonsolvated). Combined with *ab intitio* molecular dynamics, it is suggested that larger anions with lower ionic potentials (and smaller anions at greater concentrations) reduced the amount of DDAA and DA configurations, increasing free water. Therefore, anion selection for nearly neutral pH should aim for a balance that allows for efficient activation and proton transfer at the electrode/electrolyte interface but avoids excess rigidity or weakness in the hydrogen bonding structure.

Exploring this idea that anions can greatly alter interfacial properties, Liu et al.^85^ studied a complex system where two different, interacting anionic species were present in the electrolyte (Figure 5). Fluoride was added to borate buffer (pH 7.87) using an optimized Co­(OH)_2_ OER catalyst. An effect was noted between the anion and borate species that was not only additive in nature but rather dynamically synergistic; a specific hierarchical arrangement of the anions at the electrolyte-electrode interface seemed to bolster the OER current (Figure 5a,c,d). SERS and molecular dynamic simulations revealed that borate anions occupied the space closer to the electrode and fluoride anions extended outward. The advantage of such positioning is suggested to stem from fluoride’s disruption of the hydrogen-bonding network. This allows borate species to penetrate closer to the surface, generating a more compact double layer and providing proton-relaying services (Figure 5a,b). Most importantly, this compaction might greatly change the local electronic field, which is thought to increase the self-diffusion of water molecules. This leads to water molecule activation as seen by mean square displacement. The benefit of this synergy also showed lower charge transfer resistance, which was noted in electrochemical impedance spectroscopy (EIS) measurements. Comparative EIS measurements at 1.3 V (vs SHE) and 1.45 V (vs SHE) of borate, fluoride and the mixture, along with the two unique Tafel slopes of Co­(OH)_2_ in K-borate/KF indicated two potential ranges thought to be characterized by either borate (lower potential) or fluoride (higher potential). Together, it is suggested that borate facilitates the OER by encouraging local PCET, and fluoride pushes borate closer to the surface at higher potentials, aiding water activation and creating larger current densities. Experimentally, this resulted in Co­(OH)_2_ in mixed borate/fluoride at 1.4 V vs RHE outperforming pure borate or potassium fluoride media by 7.24 and 9.49 times in measured current density, respectively. Hao et al.^89^ showed similar results with an IrO_*x*_/Ni­(OH)_2_ catalyst with the synergetic pair of borate and fluoride added to a carbonate base (pH 8.35). The activity density was ∼21-fold higher than carbonate buffer alone, and the Tafel slope became singular, ∼17 mV dec^–1^ lower than the smallest Tafel slope of the three observed in the carbonate buffer.

**Figure 5:**
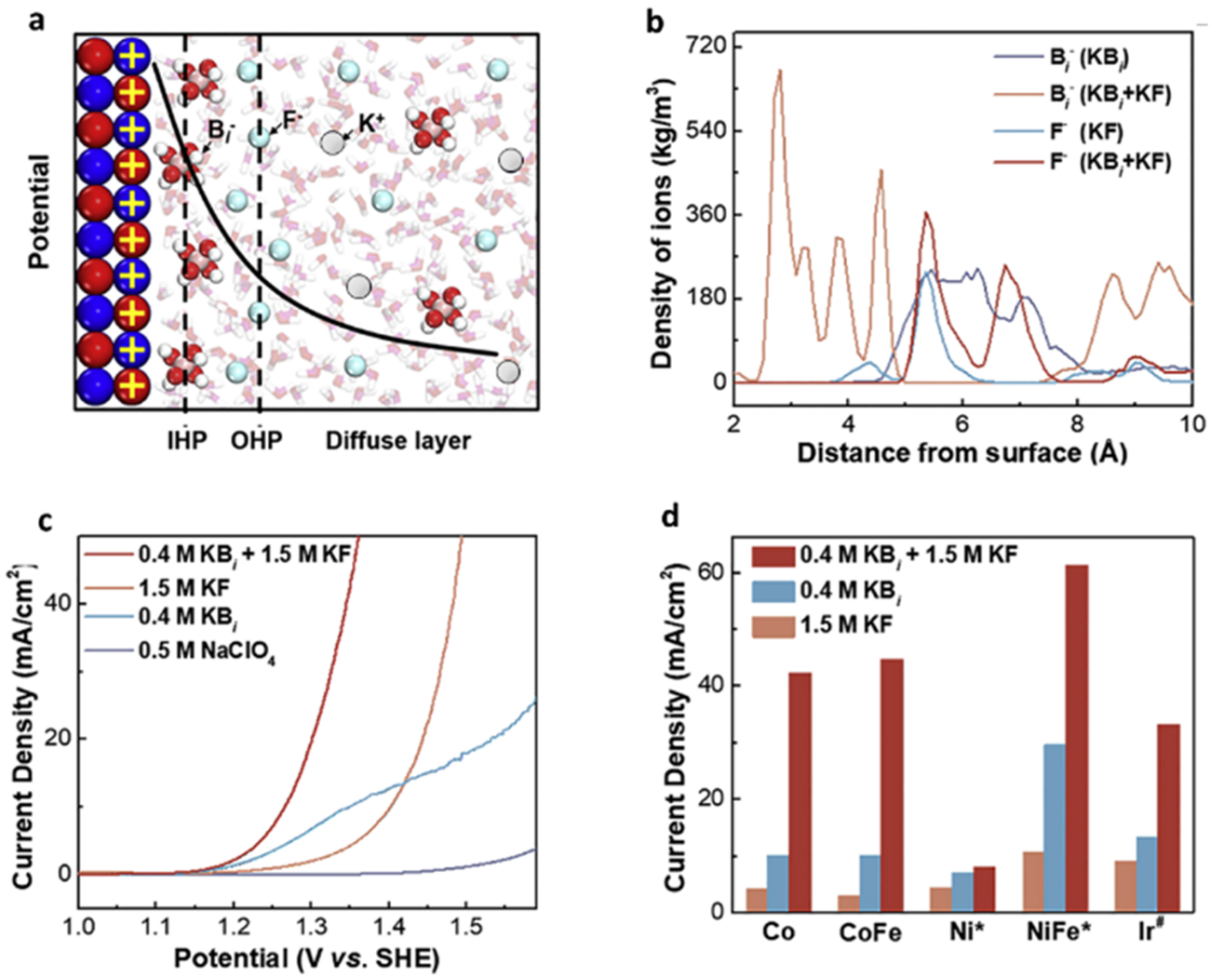
Synergetic effects of fluoride and borate in neutral electrolyte. (a) The electrode–electrolyte interface hierarchical arrangement of borate and fluoride through molecular dynamics. (b) Double layer anion distribution depending on species present and order added. (c) Electrodeposited Co­(OH)_2_ LSV measurements in different electrolytes. (d) Current densities collected at 1.35 V for different electrode materials in different electrolytes. All panels (a), (b), (c), (d) are reproduced with permission from ref 85 copyright @ 2022 Elsevier.

Zhao et al.^26^ went one step further using the same catalyst (Co­(OH)_2_) and probed into which specific borate species was causing the synergistic effect. Noting similar effects to the hydrogen bonding structure and charge transfer resistance as Liu et al.,^85^ they employed nuclear magnetic resonance (NMR), mass spectroscopy (MS), and Raman spectroscopy to examine the speciation that resulted from adding KF to borate buffer. This approach aimed to identify the anionic species responsible for the beneficial effects elevating the OER. One such species that could be attributed to increased performance is BF_2_(OH)_2_^–^; it exhibits good buffering capacity and RDS proton-accepting behavior, as shown by DFT calculations. However, an optimal amount of KF in the electrolyte was noted. Even though BF_2_(OH)_2_^–^ increases with KF addition, beneficial effects for the OER waned after a 1:3 KF to borate concentration in a volcano-like dependency. This decrease is thought to be a consequence of the increasing concentration of other species like BF_4_^–^ and BF_3_(OH)^−^. BF_4_^–^ and BF_3_(OH)^−^ are proposed to have limited buffering capacity and thwart the OER through active site inactivation as measured by Co^2+^/Co^3+^ peak areas, respectively.

The discovery of a strong synergetic interaction between fluoride and borate presents a new avenue and promising strategy for OER enhancement in pH-benign conditions. This research emphasizes the importance of electrolyte engineering to optimize electrocatalytic performance, moving beyond catalyst development as the sole focus. However, exploration thus far has been limited to a small subset of catalyst systems. Given that the electrode/electrolyte interface varies greatly depending on the catalyst, a systematic study across catalyst families is warranted to elucidate trends in how the synergetic duo interacts with different surfaces.

### 4.2 How Can Cations in Nearly Neutral Electrolytes Improve the OER?

Moreover, while recent studies have highlighted OER improvement through anion addition, cationic species in the electrolyte can enhance catalysts by integration into the surface. One example is the intentional or accidental incorporation of Fe^3+^ into electrolytes, which has long been explored for the alkaline OER using Ni-based catalysts.^90,91^ Noticeable OER improvements from Fe contaminants have been proposed to stem from Fe-enhancing conductivity, changing the local electronic environment, and activating a partial charge transfer effect on Ni.^91,92^ Anantharaj et al.^90^ nicely outline the effects seen in alkaline environments in their review. However, the quasi-neutral environment presents distinct thermodynamic complexities that must be clarified. In a nearly neutral environment, Ni and Fe at 25 °C in (bulk) pH 7 exhibit thermodynamic instability as seen in Beverskog et al.’s Pourbaix diagrams.^93,94^ They are thermodynamically prone to dissolution and disposed to existing predominantly in ionic form, which contrasts with the stable solid oxyhydroxide phases in alkaline media. Thus, the electrolyte is imperative to catalyst performance in quasi-neutral pHs: both the pH (with the buffering capacity in mind) and the species present. As mentioned in Section 3.2, quasi-neutral electrolyte species can stabilize a catalyst, prevent dissolution, and unlock part of the benefits of Fe seen in alkaline conditions. For example, borate is proposed to stabilize Ni-based catalysts via the formation of a complex oxide.^74^ Smith et al.^95^ investigated the impacts of Fe^3+^ on NiO_*x*_-Bi films in these stabilizing borate buffer conditions (pH 9.2). Their study examined films conditioned by applying a sustained potential at 0.856 V vs SCE for various times (up to 130 min) with interval CVs in either Fe-free, 0.1 or 1.0 ppm Fe^3+^ contaminated 0.5 M borate buffer. XPS was used to characterize the relative amount of Fe incorporated into the film surface. Films conditioned with 1 ppm Fe for 2 h had 14% Fe relative to the Ni content. Those conditioned in 0.1 ppm Fe had 5% Fe relative to Ni. These results were then compared to CV measurements. Films conditioned in Fe-free electrolytes did not seem to improve in either current density or onset potential. Activity improvement aligned with increasing iron content. Furthermore, Tafel plots showed similar mechanistic principles backed all experimental catalysts, but Fe-conditioned or Ni–Fe codeposited films had ∼30 and ∼20 mV dec^–1^ lower Tafel slopes than Fe-free catalysts. While films conditioned in higher Fe content exhibit similar behavior to codeposited films, the idea that the reconstruction of catalysts can be facilitated by electrolyte species is imperative. This underscores that the constituents present in the electrolyte are critical variables in the dynamic development of catalysts, and these variables can be harnessed in neutral environments, where the OER faces major challenges.

## 5 What External Factors Can Enhance the OER in a Quasi-Neutral Environment?

In addition to electrolyte additives, external system parameters can greatly affect the OER performance and can be easily applied to improve the neutral electrolyte environment. Increasing the diffusion coefficient (*D*) (Stoke’s equation) and the standard rate constant (*k*_0_) (Arrhenius’ equation) is obtained by adding more energy into the system in the form of temperature:^66^

2 $$D=\frac{k_{B}T}{3\pi d\mu }$$

3 $$k_{0}=A^{\prime }e^{-E_{a,\mathrm{app}}/\mathrm{RT}}$$

where *k*_B_ is the Boltzmann constant, *T* is the temperature in Kelvin, *d* is the hydrated ion’s effective diameter, μ is the solution viscosity, *A′* is a preexponential factor, *E*_a,app_ is the apparent activation energy and *R* is the gas constant. Temperature increase results in a larger diffusion coefficient, which aids mass transport, and increases the standard rate constant. Typically, water splitting with PEM and alkaline electrolyzers uses temperatures around 40–80 °C to maximize efficiency.^96^ While numerous studies have investigated increased temperatures for acidic or alkaline conditions, there is a significant dearth of information on quasi-neutral electrolyte physicochemical properties at different temperatures (like viscosity) that influence the diffusion coefficient and, subsequently, their water-splitting performance.

### 5.1 Does Increasing Temperature Enhance the OER in Nearly Neutral Electrolytes?

Beyond kinetics, changing the temperature can alter the behavior of an electrolyte, in turn impacting a system’s performance. Naito et al.^97^ investigated the neutral OER temperature dependence using a Pt cathode and IrO_*x*_ anode phosphate buffer system. Before evaluating electrochemical performance, they examined how electrolyte properties changed in the range of 25–100 °C. Their findings revealed that while K-phosphate offered an optimal combination of properties, including solubility and higher conductivity than Na-phosphate at a given concentration, allowing for a smaller ohmic drop, the most significant temperature-driven benefit was a decrease in measured viscosity with increasing temperature. This reduction in viscosity was proposed to be the primary influence on mass transport (with variation in the mean activity coefficient and hydration numbers being comparatively smaller). Using this insight, they tested 0.1 and 3.5 mol kg^–1^ K-phosphate buffer solutions at 80 °C in a three-electrode configuration and a full electrolyzer cell. At this temperature, a full cell with phosphate solutions showed higher faradaic efficiency than acidic (HClO_4_) or alkaline (KOH) systems. This was attributed to less Ir dissolution, where a self-healing mechanism in quasi-neutral electrolytes described in a separate paper by Kanan et al.^87^ could be active. While dissolution or IrO_*x*_ in acidic medium was unexpected, this was ascribed to the direct contact of acidic H_2_O with the anode, where, compared to a PEM electrolyzer, pure H_2_O is fed and H_3_O^+^ is directly transported through a solid electrolyte. In alkaline media, this finding was also supported as the overpotentials of the IrO_*x*_/Ti mesh-Pt/Pt system relatively matched that of the bare Ti mesh-Pt/Pt mesh in KOH over time, indicating a loss of the active IrO_*x*_ layer. Remarkably, it also had better stability with on/off cycling and continuous cycling. That said, K-phosphate needed 1.49 V at 100 °C to obtain 100 mA cm^–2^ while alkaline solutions needed 1.5 V at 73 °C.

Building on the impact of temperature for buffered systems, a study by Nishimoto et al.^66^ emphasizes the interplay of electrolyte choice and temperature. They tested a 1.5 mol kg^–1^ carbonate solution (pH 10.5) and a NiFeO_*x*_ catalyst at 80 °C. Their findings revealed a notable temperature-dependent difference in onset potential between alkaline and buffered conditions. At 30 mA cm^–2^, the onset potential differed by 151 mV at 25 °C and shrank to 18 mV at 80 °C. This improvement most likely reflects *E*_a,app_. However, at higher overpotentials, a noticeably higher Tafel slope of ∼60 mV dec^–1^ was proposed to be caused by carbonate surface coverage. Even with this disadvantage, the carbonate system’s exchange current density allowed the systems to be comparable at increased temperatures, showing similar fundamental catalytic activity to that of alkaline electrolyte. This emphasizes the critical importance of understanding the electrolyte-electrode interaction, as differences in overall OER performance stem from environmental effects on both mass transport and reaction kinetics. Nonetheless, a practical consideration for these systems is the long-term stability of (bi)­carbonate buffers at elevated temperatures, which requires careful monitoring and characterization due to their known tendency to release CO_2_, which can result in a pH shift to higher values. Therefore, while results show that elevated temperatures enhance the OER performance for neutral systems and allow them to be more comparable to acidic/alkaline conditions, a lack of physicochemical information for neutral electrolytes remains a hurdle for the progression of the OER in nearly neutral electrolytes.

### 5.2 How Do We Improve Mass Transport in Nearly Neutral Environments?

Mass transport is mainly determined by the electrolyte properties as named above. As buffers tend to have a higher viscosity than other salt-based electrolytes, mitigating mass transport effects is difficult. Buffer species must leave the interface after protonation and unprotonated species must come to the interface. Mass transport is described in three parts within the one-dimensional Nernst–Planck equation:

$$J_{i}\left(x\right)=-D_{i}\frac{\partial c_{i}\left(x\right)}{\partial x}-\frac{z_{i}F}{\mathrm{RT}}D_{i}c_{i}\frac{\partial \phi \left(x\right)}{\partial x}+c_{i}\nu \left(x\right)$$ 4

where *J* is flux, $\frac{\partial c_{i}\left(x\right)}{\partial x}$ is the concentration gradient, *D*_*i*_ is the diffusion coefficient for particle *i*, *z*_*i*_ is the charge number, *F* is the Faraday constant, $\frac{\partial \phi \left(x\right)}{\partial x}$ is the electrical potential gradient and *ν­(x)* is the hydrodynamic velocity. The first term relates to diffusion, the second describes migration, and the last encompasses convection. Typically, rotating disk electrodes help control the contributions from mass transport, allowing the reaction to become diffusion-limited and enabling the proper examination of the kinetic properties of electrocatalysts. While rotating disk electrode setups are quite effective for extreme pH conditions and can maintain a thin diffusion layer thickness at lower rotation speeds, mass transport can be debilitating to the neutral OER, especially noted around pH ∼ 11 due to reactant switching (as discussed Section 2, Figure 1b); rotation speeds must be increased to allow for the diffusion of OH^–^. For example, Shinagawa et al.^25^ evaluated Ni and Co disk electrodes in 0.1 M KOH (12.8 pH), 0.5 M K-sulfate (pH 10.8), K-carbonate (pH 10.8), and K-phosphate (pH 9.4) for diffusion layer properties. In alkaline conditions, Ni and Co RDE measurements showed little dependency on rotation rate at potentials corresponding to the OER. In an unbuffered solution (pH 10.8), activity increased with higher rotation speed, indicating dependency on the thickness of the diffusion layer and thus reliance on diffusion of OH^–^ ions. Interestingly, in K-phosphate, Shinagawa et al. also noted that reactant switching was not apparent and there was dependency on rotation speed. Ultimately, this study concluded that the diffusion of the protonated buffer species impacted and limited the OER performance. Compared to the HER, which is hindered by the diffusion of deprotonated buffer species, the OER performance increases with buffer concentration. This indicates, while not definitively, that the diffusion of protonated species could be the limiting factor. As the diffusion layer thickness is proportional to diffusion and rotation speed, ensuring rotations are high enough to combat large concentration gradients and stagnation is crucial for obtaining meaningful kinetic data.

While the rotation speed only controls the convective mass transport, the electrolyte species and their properties dominate diffusive mass transport; the identity, mean activity, size, and molality of the electrolyte species influence diffusive mass transport and the OER performance. Shinagawa et al.^25^ studied NiO_*x*_ and CoO_*x*_ catalysts in various mild pH buffers using RDE at 3600 rpm. They noted that when the concentrations of buffer species increased up to 3.0 kg mol^–1^, the overpotential required to achieve 10 mA cm^–2^ decreased monotonically. This was surprising considering the link between viscosity and performance discussed in Section 5.1. This contrasts with the HER, where the concentration and overpotential relationship forms a volcano-like plot. It was suggested that the increased availability of buffering species involved in proton management affects the OER and outweighs the reduced diffusion coefficient from raised concentrations. Other important factors that directly impact diffusion, as emphasized by Shinagawa et al., are the size of the protonated species and the viscosity of the electrolyte; less focus was put on the mean activity coefficients, as the diffusion of the protonated species is much lower in concentration when compared to unprotonated species. This finding emphasizes that electrolyte engineering holds the key to improving the OER by maximizing mass-transport flux, but additional methodical studies are needed to help elucidate trends, including those with concentration. As a proof of concept, Shinagawa et al. ran a water electrolysis cell with NiMo and CoO_*x*_ as the cathode and anode, respectively. Increasing the concentration of K-carbonate (pH 10.1) showed improvement from 1.9 V with 0.1 M to 1.7 V with 1.5 M to achieve a current density of 10 mA cm^–1^ with a reported stability >20 h (although still inferior to 0.5 M KOH). This finding is especially pertinent to the discussion around concentration, as many groups use concentrations around 0.1 M with little explanation.^11,66^ With this in mind, understanding catalyst interactions with the buffer species is of utmost importance, as increasing the species that can deactivate the surface can be detrimental.

## 6 Direction/Outlook

In this perspective, we provide a summary of the challenges associated with the nearly neutral OER and discuss current strategies in electrolyte engineering as reported in the literature. While some catalysts demonstrate excellent performance in alkaline conditions, their activity, overpotential, and stability significantly suffer in nearly neutral media. Many studies have examined how to augment OER performance by modifying catalyst properties; however, as highlighted in this perspective, this approach addresses only one part of the system interface. The performance and stability of catalysts in quasi-neutral environments are also fundamentally governed by electrolyte properties. Therefore, it is essential to consider electrolyte parameters to optimize the OER in nearly neutral electrolytes (Figure 6). Electrolyte considerations when working in a neutral environment should encompass:

- Minimizing local pH fluctuation. In quasi-neutral media, water oxidation results in an acidic environment. This can trigger catalyst dissolution, concentration gradients, and alter properties at the electrode/electrolyte interface, affecting the mechanistic and kinetic principles. Buffering electrolytes can be utilized to counteract extreme local pH swings. Buffers must be correctly chosen to maximize buffering capacity (where p*K*_*a*_ is equal to pH) where the corresponding pH value does not negatively impact the OER through favored sluggish intermediates. This relationship is also dependent on the current density.
- Examining surface interactions between the electrolyte species and the catalyst. Buffering species and cations/anions alter the fundamental properties of the EDL and interact with the catalyst surface. Buffering species can increase the catalyst’s sturdiness through adsorption. However, they can also deactivate and poison active sites, decreasing the OER performance. Therefore, evaluating the effect of buffer concentration on OER performance and catalyst stability is imperative when choosing a nearly neutral electrolyte. Moreover, in alkaline conditions, studies suggest that cations can benefit the OER by stabilizing intermediates, changing the hydrogen bonding network, and indirectly affecting pH. In neutral environments, while the pH is typically much closer to the IEP, this concept is much less studied, and systematic experiments (including studies around the more dynamic PZTC) could provide insights that could elevate OER performance.
- Enhancing OER performance via additives. Alterations of the electrode/electrolyte interface through additional additives can benefit OER performance. Adding salts can increase the conductivity of the electrolyte, change the local electric field at the interface, and change the hydrogen-bonding dynamics. Research into synergetic mixtures (borate and fluoride) has provided promising results for improving the OER in neutral conditions. This idea offers an encouraging strategy to enhance a neutral OER system beyond its current capabilities, and studies with different catalyst groupings can provide additional insight.
- Maximizing mass transport effects and thermodynamic efficiency. While buffering species can help mediate local pH, ensuring adequate mass transfer is challenging. This problem arises from the physicochemical properties of buffer solutions; trade-offs between the viscosity and the buffering capacity (increasing concentration) can hinder the diffusion coefficient. Understanding the trade-offs of physicochemical parameters and the volcano trends between them is critical for boosting the quasi-neutral OER. Enhancing mass transport, for example, by increasing the rotation speed for fundamental RDE studies or creating high flow through an electrolysis cell, may be used to improve mass transport.

**Figure 6:**
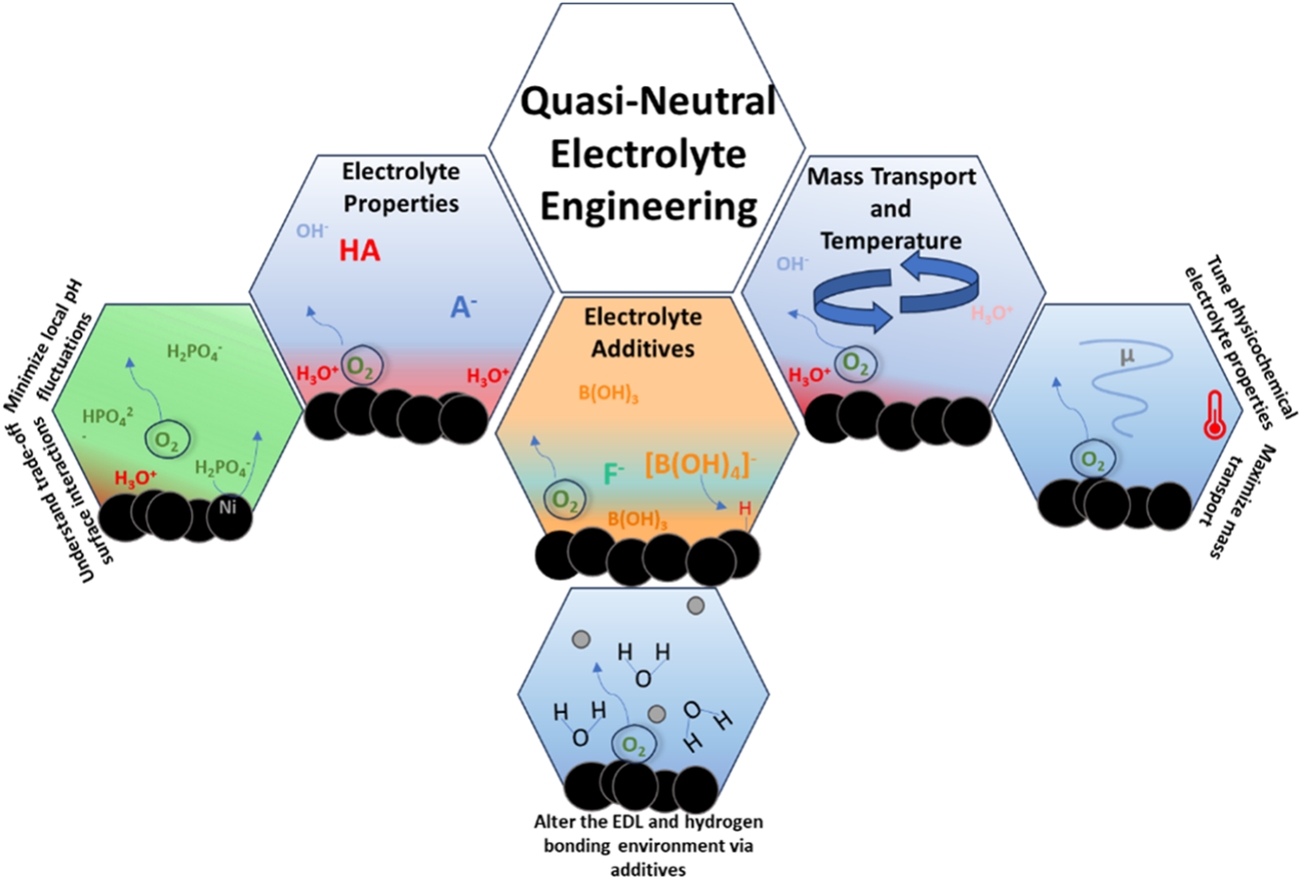
Sketch summarizing important attributes to consider when choosing an electrolyte for the quasi-neutral OER.

Beyond understanding and optimizing the catalyst/electrolyte interface to improve the quasi-neutral OER, there are profound engineering challenges in implementing nearly neutral media beyond the laboratory environment. The transition to industrial operation alters the working conditions; high current densities result in high flux of oxygen gas, requiring setups like gas diffusion electrodes and designed mass transport porous layers. Furthermore, the use of nonpure water feedstocks, which would remove the need to use scarce fresh water, poses massive problems regarding side reactions (i.e., ClER), membrane fouling, and precipitation reactions. Consequently, after efforts have been made to understand the quasi-neutral system from an activity-property relationship, future research must prioritize cell architectures that provide industrial relevance while also considering the effects of the water source. By tackling these challenges, quasi-neutral electrolysis can present an opportunity for a safer and sustainable way of producing green hydrogen.
